# Blockchain driven trust management for intelligent transportation systems in VANETs

**DOI:** 10.1038/s41598-025-23351-x

**Published:** 2025-11-12

**Authors:** Hariharasudhan V, Vetrivelan P, Elizabeth Chang

**Affiliations:** 1 School of Electronics Engineering, Vellore Institute of Technology, Chennai, Tamil Nadu 600127 India; 2 University of Sunshine Coast, Sunshine Coast, Australia

**Keywords:** VANETs, Blockchain, Enhanced QPBFT, DNN, Net reliability, RSU, Energy science and technology, Engineering, Mathematics and computing

## Abstract

Vehicular Ad Hoc Networks (VANETs) are a key component of Intelligent Transportation Systems (ITS), enabling vehicle-to-everything (V2X) communication to improve traffic efficiency and road safety. However, maintaining strong network performance and user privacy requires robust security mechanisms. Unlike existing blockchain-VANET solutions that address isolated aspects, our framework uniquely integrates Physical Unclonable Functions (PUFs) for lightweight authentication, an Enhanced Quantified role-based Practical Byzantine Fault Tolerance (EQPBFT) consensus mechanism for efficient and secure block creation, and a Deep Neural Network (DNN)-based intrusion detection system into a unified trust management pipeline. The EQPBFT algorithm improves network resilience by efficiently identifying and mitigating malicious nodes during block creation. In addition, a DNN-based intrusion detection system enhances threat classification and strengthens attack detection accuracy. Simulation results demonstrate reduced computation time (up to 25% lower than PBFT), faster block creation (5.2 ms vs. 6.6 ms for 200 nodes), and higher intrusion detection accuracy (95%) compared to baseline models. These quantitative outcomes validate the robustness and efficiency of the proposed system.

## Introduction

**Fig. 1:**
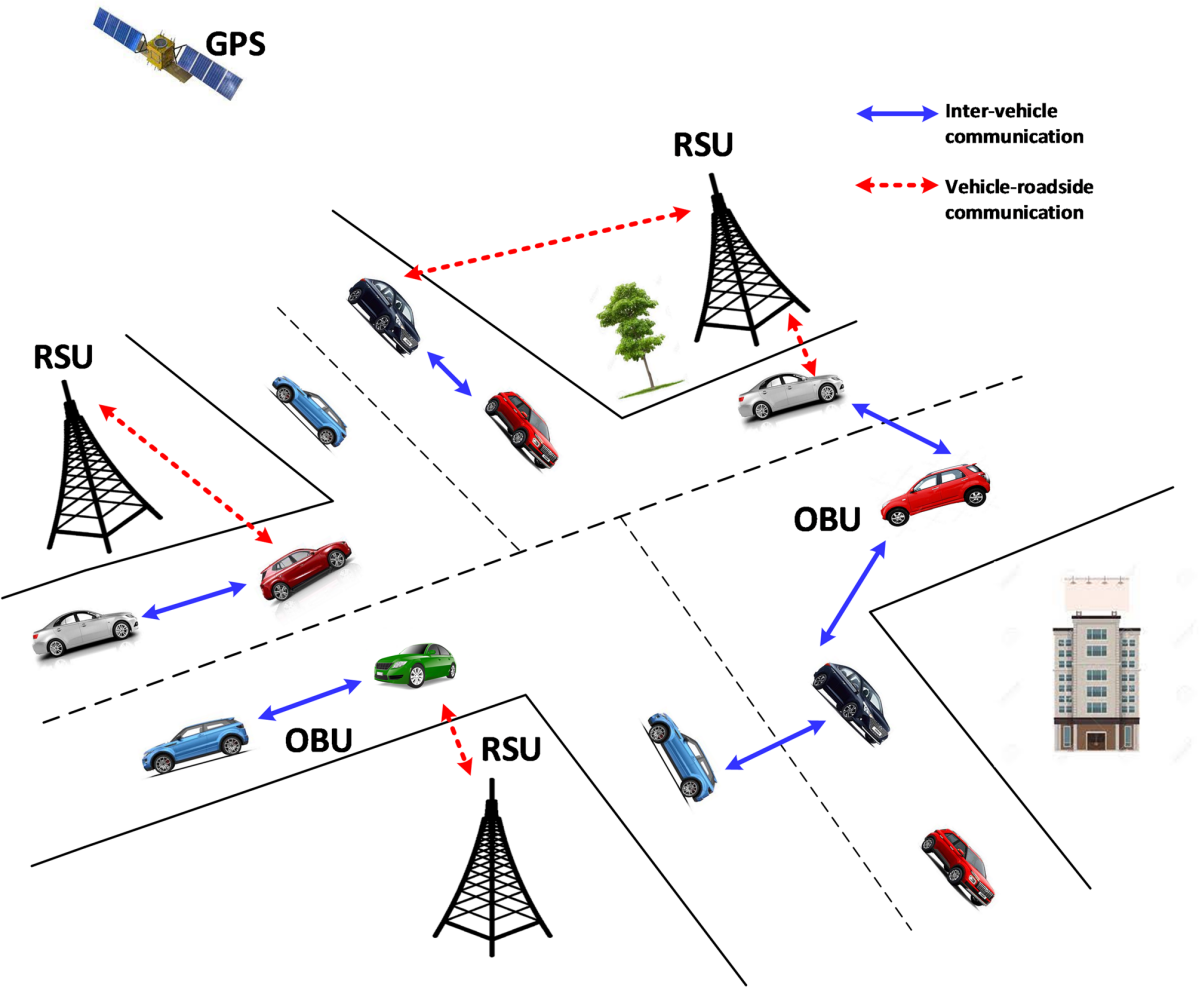
Architecture of VANETs.

Nowadays, road safety is considered essential due to the manufacturing of numerous vehicles. ITSs are crucial for modern industries, revolutionising transportation efficiency and safety through data-driven technologies. These systems optimise traffic flow, reduce congestion, and enhance road safety using real-time data and communication networks. Security in ITS is paramount to protect against cyber threats that could disrupt critical infrastructure and compromise sensitive information. Robust encryption, authentication protocols, and intrusion detection systems are essential to safeguard ITS from cyberattacks. As ITS becomes more interconnected and reliant on digital systems, ensuring robust cybersecurity measures is imperative for its continued industrial significance and societal benefit. VANETs are a wireless medium used to communicate between vehicles and between a car and an RSU. In addition to providing a wealth of information to drivers and passengers, this mode of communication enables safety applications to improve road safety and provide a more comfortable driving experience. A typical VANETs consist of an RSU, an On-Board Unit (OBU), radars, and wheel rotation sensors that employ IEEE 802.11p as a wireless Local Area Network (LAN). Figure 1 illustrates the architecture of VANETs with V2V and V2I communication.

Current technology has improved in transportation to provide information about road events by VANETs^1^. Hence, VANETs are essential to intelligent transport systems and other connecting devices that exchange data through the wireless medium in an open space environment^2^. In VANETs, vehicles communicate with each other using a Global Positioning System (GPS), Wireless Fidelity (Wi-Fi), a driving recorder, and other value-added service equipment. If any event occurs in an area, the vehicle near the event sends information to each vehicle and a query message to the RSU^3^. Traffic and safety information are transferred to nearby cars and RSUs to increase the awareness of driver and passenger safety in a given route, and the exchange of information among different nodes is an essential application of VANETs^4^. In an open network environment, malicious nodes can access the network at any moment and launch various assaults, such as Denial of Service (DOS)^5^, message suppression, and the propagation of fraudulent messages that can negatively impact the network performance.

In VANETs, the reliability of the network depends on the node. Thus, the reliability of every vehicle with RSU interactions is necessary to influence the trust evaluation mechanism that depends on this data^6^. As a result, trust management is regarded as an effective method for addressing trust and privacy issues in VANETs^7^. The RSU often evaluates the distributed trust management architecture of VANETs, which has better efficiency for problems^8^. However, maintaining trust within its transmission range is primarily the responsibility of each RSU.

Moreover, several problems have occurred in a complicated VANET environment, such as a delayed synchronisation of trust data between RSUs, significantly affecting trust calculation applications. Then, the consensus mechanism method allows RSUs to collaborate and keep a reliable database to ensure the integration of reliability evaluations. Trust evaluation and reputation calculation are critical to effectively identifying malicious vehicles in this environment.

Blockchain is the best solution to maintain the trust evaluation for vehicles because of its significant characteristics, such as consistency, fault tolerance, decentralisation, and tamper-proof^9^. Thus, this system provides enhanced capabilities and information transparency to avoid malicious attacks. The Blockchain mainly stores the information in blocks chained together, which cannot be updated or deleted^10^. This system is more robust and immutable due to adding blocks to the chains, which can only add a block at the end of the chain. Though the techniques above increase the reliability of the blockchain network to some level, malicious nodes may still engage in the consensus mechanism. The malicious node detection technique must be combined with further improvements in consensus performance. In this designed model, a deep learning-driven Blockchain-based trust management system for VANETs communication is intended for trust management and malicious node detection. Unlike existing blockchain-based security frameworks for V2X communication focusing on identity management or transaction integrity alone, our proposed model introduces a multi-faceted trust management architecture. It combines PUF-based physical identity authentication, Bayesian-based message reliability computation, an optimized EQPBFT consensus mechanism adapted for vehicular mobility, and a deep learning-based intrusion-type classifier. This layered integration enhances the trust evaluation fidelity and detection of nuanced attack types, offering a more comprehensive solution for V2X networks. Significant contributions of the proposed model are.

- Development of a blockchain-based trust management system tailored for VANETs communication, integrating deep learning techniques to enhance security and reliability.
- Introduction of a novel approach where VANET data collected from nodes is processed within Road Side Units (RSUs) to ascertain net reliability and trust values of individual nodes.
- Implement an Enhanced Quantified role-based Practical Byzantine Fault Tolerance (EQPBFT) algorithm for block creation, distinguishing between malicious and non-malicious nodes based on trust evaluations.
- Deployment of a Deep Neural Network (DNN) model to analyse malicious nodes, identifying intrusion/attack types and enhancing the system’s ability to mitigate threats effectively.

The upcoming portions of the article are organised as Sect. 2, which consists of various research papers related to VANETs, and Sect. 3 briefly explains the proposed methods. Section 4 illustrates the experimental results for the designed model, and Sect. 5 consists of a conclusion for the entire research work.

## Literature survey

Several research efforts have explored Blockchain and machine learning applications for securing Vehicular Ad Hoc Networks (VANETs), focusing on Authentication, intrusion detection, and trust management. However, many of these works treat these components in isolation and lack an integrated trust architecture tailored for the dynamic and resource-constrained nature of VANET environments.

Dwivedi et al.^11^ proposed a blockchain-enabled IPFS-based authentication framework for secure event data storage without relying on centralised cloud infrastructure. While their method supports decentralised storage and access control, it does not address trust computation or malicious node detection in real-time communication.

Chougule et al.^12^ introduced a Multibranch Reconstruction Error (MBRE)-based intrusion detection using CNNs on F-I-M (Frequency, Identity, Motion) data branches. Although this structure captures vehicular behaviour, it lacks integration with Blockchain or trust scoring mechanisms, which limits its reliability against spoofing or collusion attacks.

Vitalkar et al.^13^ employed a Deep Belief Network (DBN) for intrusion detection, demonstrating improved accuracy over traditional ML methods. However, the work primarily focuses on data classification and does not incorporate decentralised trust validation or consensus models needed for a secure VANET.

Liu et al.^14^ developed a blockchain-based collaborative IDS that offloads training to vehicular edge devices. While this reduces server dependency and preserves model privacy, their approach does not address real-time attack type identification or dynamic trust adjustment.

Yang et al.^15^ proposed a Proof-of-Event (PoE) consensus algorithm to replace conventional blockchain consensus mechanisms like PoW or PoA in VANETs. Despite its suitability for vehicular data validation, the model lacks a learning-based mechanism for detecting behavioural anomalies or refining trust.

Bangui et al.^16^ enhanced detection efficiency using an ensemble model combining Random Forest with posterior detection. However, their solution is not integrated with any secure consensus or authentication protocol, leaving the system vulnerable to Sybil or replay attacks.

Alsarhan et al.^17^ developed an IDS using a combination of Bayesian reasoning and Dempster-Shafer theory to detect suspicious nodes. While their probabilistic fusion approach is robust, it does not include scalability measures or trust storage mechanisms using Blockchain.

Inedjaren et al.^18^ incorporated Blockchain into Optimised Link State Routing (OLSR) to improve routing integrity. Although this enhances data consistency, the system performs security operations redundantly at each node, incurring computational overhead without learning-based threat adaptation.

Zhou et al.^19–21^ explored federated learning and edge intelligence to optimise caching, energy use, and privacy in IoV and mobile edge networks. While valuable in performance and efficiency, these frameworks do not address trust quantification or attack-type detection in vehicular communication scenarios.

Despite significant advancements in VANET security through blockchain and machine learning approaches, many of the reviewed studies exhibit notable limitations. Chief among these is the lack of an integrated framework that combines Authentication, trust evaluation, and intrusion detection into a cohesive trust management pipeline. Most prior works either focus on data classification using machine learning or secure data sharing via Blockchain, but rarely both. Furthermore, existing methods often rely on centralised processing, static trust scores, or pre-defined rule-based filters, which are inadequate in dynamic and decentralised environments like VANETs. Additionally, the absence of real-time behavioural analysis, attack-type classification, or scalable consensus mechanisms limits their practical deployment in high-density vehicular networks. Only a few studies explore adaptive security models, and fewer still offer detailed performance comparisons or account for evolving threats like botnets and distributed denial-of-service attacks.

Liu et al.^22^ proposed a privacy-preserving dual reputation (PPDR) management scheme in vehicle platoons. Liu et al.^23^ developed a privacy-preserving reputation updating (PPRU) scheme for cloud-assisted vehicular networks. Sharma et al.^24^ presented, a privacy-preserving trust management (PPTM) scheme for emergency message dissemination in space–air–ground integrated vehicular networks. Zhou et al.^25^ proposed a trust cascading-based emergency message dissemination (TCEMD) model in VANETs. These contributions provide valuable insights but remain limited in offering a unified integration of authentication, consensus, and intrusion detection.

To address these gaps, the proposed framework introduces a unified and novel approach to VANET trust management. It integrates three core components: Physical Unclonable Function (PUF)-based Authentication for secure V2V identity verification, an Enhanced Quick Practical Byzantine Fault Tolerance (EQPBFT) consensus protocol for efficient and lightweight trust agreement at RSUs, and a Deep Neural Network (DNN) classifier to identify and categorise malicious nodes based on communication behaviour and message reliability. This design enables real-time detection of sophisticated attacks while maintaining low latency and high trust accuracy. Unlike previous solutions, our method captures both trust and threat dimensions through a hybrid of cryptographic validation and intelligent anomaly detection. Performance evaluations, including precision, sensitivity, and error rates, demonstrate that our model outperforms existing Blockchain- and ML-based solutions in both accuracy and robustness, making it highly applicable for secure, intelligent transportation systems.

## Proposed methodology

VANETs are needed to build an intelligent transportation system that improves traffic flow and provides event information on the road. Data confidentiality is at risk from eavesdropping, illusion, social, and traffic analysis attacks. Thus, a secure trust management VANET communication is designed based on Blockchain with intrusion-type detection.

**Fig. 2:**
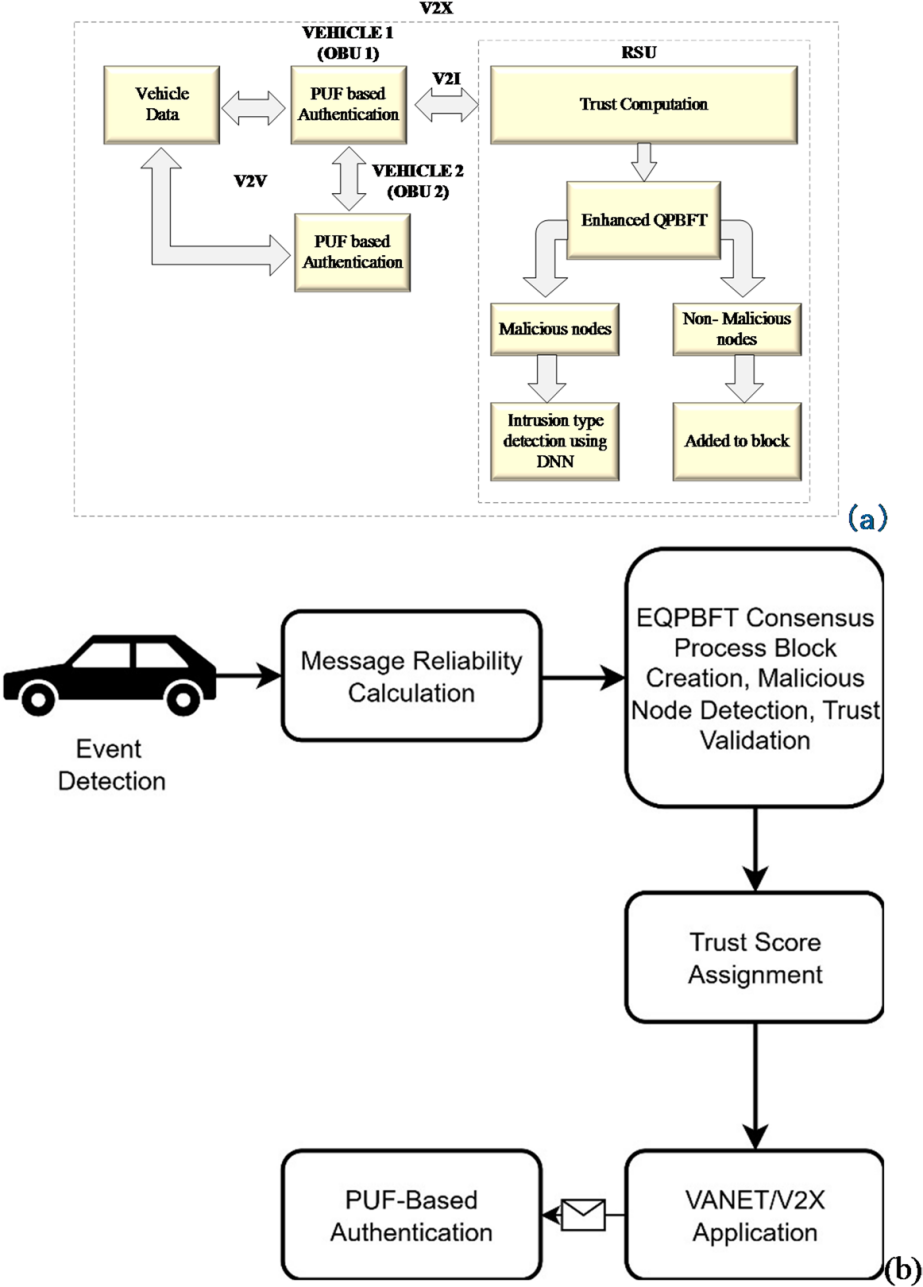
**(a)** Process flow of the designed trust management system. **(b)** Event detection to final trust score.

Figure 2a illustrates the process flow of the Blockchain-based trust management system for VANETs communication. At first, data is collected from different nodes in VANETs using vehicle data. For OBU-to-OBU transmission, PUF-based Authentication is used for transferring the data securely. Then, the shared data from the vehicle is collected and transmitted to the RSU. RSU to calculate the node’s net reliability and trust value by the sender identity and the event happening. Then, the block is generated by EQPBFT to find the non-malicious and malicious nodes based on several tasks. The non-malicious node is added to the block to store data, and the malicious node is given to the intrusion detection system to classify the type of attack using DNN.

### On-board unit (obu)

The proposed VANET architecture consists of a dynamic mix of vehicle nodes (OBUs), roadside infrastructure (RSUs), and a central cloud server, all interconnected through V2X communication.

- Vehicles and OBUs: The network includes 100 vehicle nodes, each equipped with an On-Board Unit (OBU). These OBUs are embedded with multiple sensors (GPS, accelerometers, cameras) and communication modules that support Dedicated Short-Range Communication (DSRC) and 5G.
- Vehicle-to-Vehicle (V2V): Among the vehicle nodes, 25 pairs are engaged in V2V communication simultaneously. These V2V links are primarily used for local traffic alerts, collision warnings, and cooperative driving.
- Vehicle-to-Infrastructure (V2I): The network also includes 10 RSUs strategically placed at road intersections and traffic control points. Each vehicle can establish V2I communication with RSUs to send and receive authenticated messages such as speed limits, traffic signals, or road hazard notifications.

### PUF-based authentication

Physical unclonable functions (PUFS) provide a lightweight and secure means of device authentication, particularly suitable for resource-constrained environments such as vehicular nodes in vanets. A PUF is a physical entity embedded within a hardware component that leverages inherent manufacturing variability to produce a unique and unpredictable response to a given challenge.

The authentication mechanism follows a challenge-response model. when a trusted server sends a challenge C to a vehicular node, the PUF embedded in the node processes it based on its unique hardware characteristics and returns a response R, such that:

$$\:R=PUF\left(C\right)$$

Due to the intrinsic physical variations introduced during IC fabrication, no two PUFs generate the same response for the same challenge. This makes the function practically unclonable and highly secure against spoofing or key extraction attacks. In our proposed system, PUF is used at the physical layer for secure identity verification of vehicular nodes before engaging in blockchain-based communication. This ensures that only physically authenticated vehicles can exchange messages, reducing the risk of Sybil and impersonation attacks.

PUF architectures can be broadly classified into two types:

- Strong PUFs: Capable of generating a large set of distinct Challenge-Response Pairs (CRPs), making them ideal for remote Authentication.
- Weak PUFs: Generate limited CRPs and are primarily used for on-chip key generation and storage.

By deploying strong PUFs in our system, we establish a robust challenge-response authentication protocol that is computationally efficient and difficult to replicate or forge. This forms the first layer of trust in our multi-layered trust management framework.

To improve clarity, a new block diagram Fig. 2b has been added. This diagram illustrates the complete workflow from event detection **→** message reliability computation **→** EQPBFT consensus execution, **→** final trust score assignment. The diagram also highlights the interactions among OBUs, RSUs, and blockchain components, ensuring transparency in how trust is established across the network.

### Calculate message reliability

In VANETs, vehicles communicate with each other and with Roadside Units (RSUs) over a wireless medium. Each vehicle has environmental sensors, processing units, and wireless interfaces to detect road conditions and generate event-driven messages. When an event is detected, a vehicle Vsen sends a message mmm with five components: sender ID, location L, message content m_i_, environmental data e, and timestamp t. A confidence value accompanies this message $\:{C}_{m}$, which reflects the reliability of the message based on the sender’s proximity to the event. The confidence value is calculated using Eq. (1):

1 $$\:{C}_{m}=\frac{1}{1+{e}^{s\left(g-\left({\left({D}_{s}\right)}^{-1}\right)\right)}}$$

where Ds is the distance between the sender and the event location. The value of $\:{C}_{m}$ Ranges from 0 to 1. Due to high mobility, multiple vehicles may detect the same event and transmit identical messages to the receiver. The receiver, based on sender locations, assigns confidence values. $\:{C}_{1}$, $\:{C}_{2}$,…, $\:{C}_{n}$ Forming vector $\:C$.

To determine the message reliability q(m∣O, C), we integrate:

- $\:O$: sender’s trustworthiness history for a given message type.
- $\:C$: the vector of message confidence values.

As a result, the receiver can attain net reliability by using the message reliability and confidence score of the particular message, which is represented in the following Eq. (2):

2 $$\:q\left(m|O,C\right)=\frac{q\left(m|C\right)\times\:q\left(C|m,O\right)}{\left(q\left(m|C\right)\right)\times\:q\left(C|m,O\right)+q\left({m}^{{\prime\:}}|C\right)\times\:q\left(C|{m}^{{\prime\:}},O\right)}$$

Where, $\:q\left(m|C\right),\:q\left(C|m,\:\:O\right),q\left({C}_{s}|m,O\right)$ They are given by Eqs. (3)–(5).

3 $$\:q\left(m|C\right)=\frac{q\left(m\right)\times\:{\prod\:}_{s=1}^{N}q\left({C}_{s}|m\right)}{q\left(m\right)\times\:\prod\:_{s=1}^{N}q\left({C}_{s}|m\right)+q\left({m}^{{\prime\:}}\right)\times\:\prod\:_{s=1}^{N}q\left({C}_{s}|{m}^{{\prime\:}}\right)}$$

4 $$\:q\left(C|m,\:O\right)={\prod\:}_{s=1}^{N}q\left({C}_{s}|m,O\right)$$

5 $$\:q\left({C}_{s}|m,O\right)=\:q\left({C}_{s}|O\right)$$

Where,$\:\:{C}_{s}$ Represents the independent message type $\:m$ in Eq. (5).

6 $$\:q\left({C}_{s}|O\right)=\:\frac{q\left({C}_{s}\right)\times\:q\left(O|{C}_{s}\right)}{q\left({C}_{s}\right)\times\:q\left(O|{C}_{s}\right)+q\left({C}_{s}\right)\times\:q\left(O|{C}_{s}{\prime\:}\right)}$$

In Eq. (6), it is assumed that $\:q\left({C}_{s}\right)$ Determines the normal distribution. Moreover, $\:q\left({C}_{s}{\prime\:}\right)$ denotes 1-$\:\:q\left({C}_{s}\right)$. For $\:q\left(O|{C}_{s}\right)$ and $\:q\left(O|{C}_{s}{\prime\:}\right)$ Eq. (6) is determined using Eqs. (7) and (8).

7 $$\:q\left(O|{C}_{s}\right)={\prod\:}_{s{\prime\:}=1}^{N}q\left({o}_{s{\prime\:}}|{C}_{s}\right)={\prod\:}_{s{\prime\:}=1}^{N}{o}_{s{\prime\:}}$$

8 $$\:q\left(O|{C}_{s}{\prime\:}\right)={\prod\:}_{s{\prime\:}=1}^{N}q\left({o}_{s{\prime\:}}|{C}_{s}\right)={\prod\:}_{s{\prime\:}=1}^{N}1-{o}_{s{\prime\:}}$$

In Eq. (2), $\:q\left({m}^{{\prime\:}}|C\right)$ It is determined by using Eq. (9).

9 $$\:q\left({m}^{{\prime\:}}|C\right)=\frac{q\left({m}^{{\prime\:}}\right)\times\:{\prod\:}_{s=1}^{N}q\left({C}_{s}|{m}^{{\prime\:}}\right)}{q\left({m}^{{\prime\:}}\right)\times\:\prod\:_{s=1}^{N}q\left({C}_{s}|{m}^{{\prime\:}}\right)+q\left(m\right)\times\:\prod\:_{s=1}^{N}q\left({C}_{s}|m\right)}$$

Here,$\:q\left({C}_{s}|m\right)$, $\:q\left({C}_{s}|{m}^{{\prime\:}}\right)$ and $\:q\left(m\right)$ denotes $\:{C}_{s}$, 1-$\:{C}_{s}$ and 1-q(m), respectively. Then, $\:q\left(C|{m}^{{\prime\:}},O\right)$ is derived in Eq. (10)

10 $$\:q\left(C|{m}^{{\prime\:}},O\right)=\:q\left(C|O\right)={\prod\:}_{s=1}^{N}q\left({C}_{s},O\right)$$

Substituting Eqs. (3), (6), (9), and (10) at Eq. (2) for finding the message reliability on the RSUs based on event message and confidence score.

**Algorithm:**
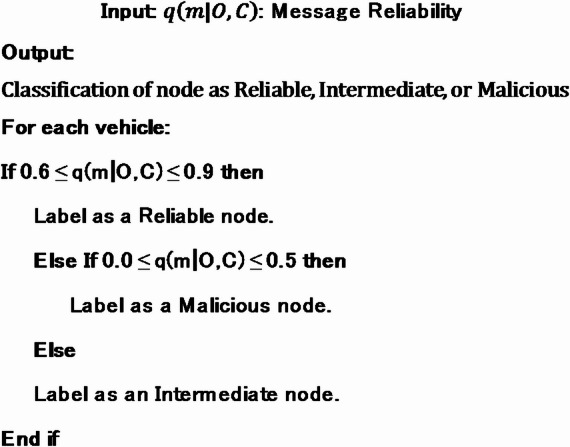
Calculation of message reliability.

In the algorithm for calculating message reliability, the thresholds of 0.6, 0.9, and 0.5 are strategically chosen to categorise nodes based on their confidence levels. A reliability value between 0.6 and 0.9 signifies messages with high confidence in their accuracy and trustworthiness, thus labelled as “Reliable nodes.” Conversely, values between 0.0 and 0.5 indicate low confidence, suggesting potential inaccuracies or malicious intent, hence identified as “Malicious nodes.” The threshold of 0.5 serves as a clear boundary, designating messages as “Intermediate nodes” when their reliability falls within this range, indicating uncertainty in their trustworthiness. These thresholds allow for effective differentiation between reliable, potentially malicious, and uncertain nodes within the vehicular network, aiding in efficient decision-making processes.

### Calculate the net reliability of the RSU

Each RSU collects multiple message reliability values q(m∣O, C) from different vehicles about a specific event. to compute the net reliability $\:O\text{v}$ of the event at the rsu level, we use the following sigmoid-based aggregation in eq. (11).

11 $$\:O{\text{v}} = \frac{1}{{1 + e^{{ - Q\left( {\left( {\frac{1}{{{\acute{R}}^{{\prime \prime }} }}} \right)\sum \: q(m|O,C) - x} \right)}} }}$$

Where $\:O\text{v}$: Net reliability score from the RSU. $\:\varvec{O}:\:$The set of observed message reliabilities received by the RSU. Q: Slope parameter. R′: Normalisation factor based on the number of messages. $\:x$: Decision threshold.

The RSU’s trust in the event increases as more reliable messages are received. When message reliability is low, $\:O\text{v}$ approaches 0, and when reliability is high and consistent, $\:O\text{v}$ approaches 1. Table 1 represents the symbols used in Equations.

**Table 1:** Definition of symbols used in equations.

Symbols	Definition
$\:m$	Event message
$\:C$	The vector sum of Cs values for message type m
$\:{o}_{s}$	The quantity of the sender’s net reliability calculated by RSU
$\:O\text{v}$	The vector sum of $\:{o}_{s}$ values
$\:N$	Total RSUs
$\:\acute{R}^{\prime \prime}$	The number of vehicles taking part in the scoring
$\:Q$	Steepness
$\:x$	The inflexion point of the S-curve
$\:{e}^{s}$	Event confidence score
$\:g$	Event location
Vsen	Sending vehicle ID
L	Location information of the sender
mi	Message content
e	Environmental/contextual data
t	Timestamp of the message
Cm	Message confidence value, range: [0,1][0,1][0,1]

### Block generation

Selecting reliable nodes to take part in the consensus is the specific strategy. The blockchain node reliability scoring algorithm serves as the premise and basis of the Enhanced QPBFT consensus mechanism. The block is generated based on the practical Byzantine fault tolerance based on the qualified role.

Enhanced QPBFT provides a voting mechanism for node selection on the network to produce blocks based on the node reliability values to strengthen the consensus algorithm’s effectiveness and network reliability. Nodes can be divided into three groups, such as reliable nodes, malicious nodes, and intermediate nodes, based on the reliability scores. Blocks can be generated by trustworthy nodes with high-reliability ratings, ensuring reliability and accuracy. Malicious nodes with the lowest reliability scores are isolated for the safety and stability of the Blockchain. Intermediate nodes determined from the low-reliability scores served as candidate nodes.

### Enhanced QPBFT

**Fig. 3:**
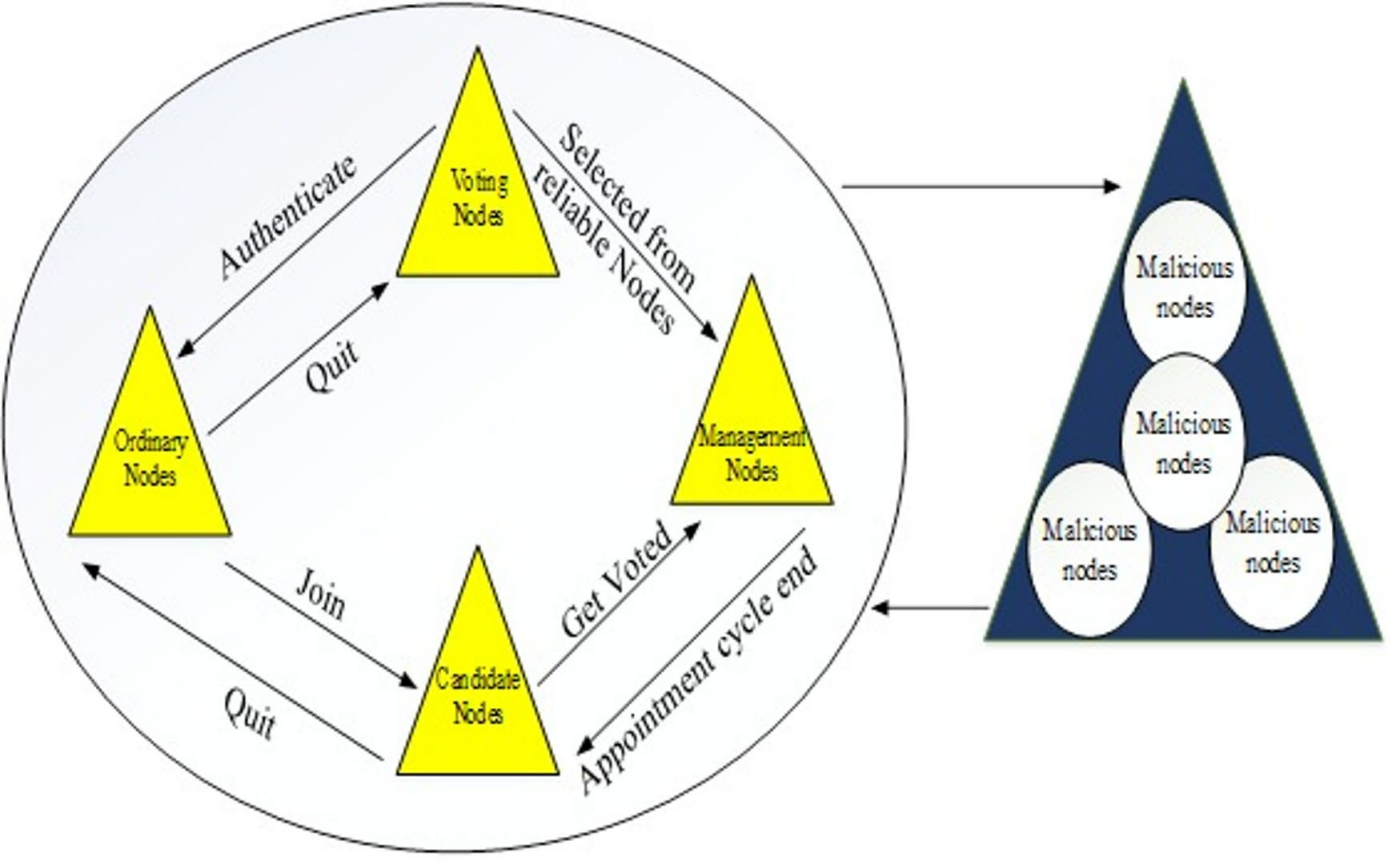
Model diagram of enhanced QPBFT.

In this Enhanced QPBFT technique, there are four categories of nodes in the network: ordinary nodes, voting nodes, candidate nodes, and management nodes. There are many nodes throughout the entire network, in which the management nodes are selected as reliable nodes based on the voting mechanism, and the malicious nodes are isolated. The model diagram of the Enhanced QPBFT is represented in Fig. 3.

From the attained reliability values of the nodes, the appointment cycle end should be updated at each round for votes. The reliability score of the node constantly alters the four different node roles to accommodate active networks and enhance the performance of the consensus algorithm. The voting nodes can verify the created transactions of the management node for voting and recommendation. Voting nodes are generated by using real-name Authentication from regular nodes. Then, by receiving a minimum of $\:\frac{{N}_{s}}{2}+1$ nodes during specific appointment cycles, the management nodes can construct blocks. The voting nodes can leave or join at any time, which is the source of the candidate nodes.

The proposed EQPBFT algorithm enhances traditional PBFT by incorporating a probabilistic voting mechanism that selects management nodes based on real-time reliability scores. This design:

- Reduces the risk of Sybil attacks by filtering low-trust nodes.
- Achieves faster consensus under dynamic traffic and attack conditions.
- Improves scalability via linear message complexity, unlike the quadratic growth in PBFT.

### Steps involved in enhanced Qpbft

The proposed intrusion detection system employs a deep neural network (DNN) trained on multi-class attack data specific to V2X environments. Unlike prior works that use static anomaly detection or rule-based techniques, our DNN model adapts to new patterns and significantly improves accuracy, precision, and specificity, which are validated on benchmark V2X datasets. Malicious nodes are immediately segregated and cannot interact with others due to node filtering. This node filtering technique removes the malicious nodes based on the attained node reliability score, which ranges in the blockchain transaction as {i|0.6$\:\le\:{O}_{V}\le\:1$}. For the preparation of each node, a unique ID is given for all the nodes. Then, the node management uses ID (K) transmissions for data block messages and production block requests to the voter.

Here, k is a random number, 0 for the genesis block. Once the incoming data block is still not maliciously altered, the voting node checks for it. After the time stamp and signature have been added, the data block message will be returned as confirmation. After getting voters’ approval messages, the management node generates the data blocks. If the block creation fails at the predetermined time by the management node, then a suitable re-election will be carried out. Candidates for management nodes are chosen from the block-producing candidate nodes based on their reliability.

To select the management node, the probability ($\:P$) of candidate nodes obtaining votes ($\:y$) is calculated. This is determined by using some of the processing factors, such as the number of voting nodes ($\:{N}_{s}$) and candidate nodes ($\:{N}_{d}$) as well as the total votes by each voter ($\:x$). The probability of choosing the management node is determined using Equation (12).

12 $$\:P={C}_{{N}_{s}}^{y}\times\:{\left(\frac{x}{{N}_{d}}\right)}^{y}\times\:{\left(1-\frac{x}{{N}_{d}}\right)}^{{N}_{s}-y}$$

13 $$\:\frac{{N}_{s}!}{\left({N}_{s}-y\right)!y!}\times\:{\left(\frac{x}{{N}_{d}}\right)}^{y}\times\:{\left(1-\frac{x}{{N}_{d}}\right)}^{{N}_{s}-y}$$

To make the voting mechanism more effective, the management node should attain more node votes than $\:\frac{{N}_{S}}{2}$. $\:{P}_{C}$ Denotes the probability that the candidate node will receive more node votes than$\:\frac{{N}_{S}}{2}$.

14 $$\:{P}_{C}=\sum\:_{y=\frac{{N}_{s}}{2}+1\:}^{{N}_{s}}P$$

15 $$\:\sum\:_{y=\frac{{N}_{s}}{2}+1\:}^{{N}_{s}}\:\frac{{N}_{s}!}{\left({N}_{s}-y\right)!y!}\times\:{\left(\frac{x}{{N}_{d}}\right)}^{y}\times\:{\left(1-\frac{x}{{N}_{d}}\right)}^{{N}_{s}-y}$$

Hence, the obtained nodes should exceed $\:{N}_{s}$/2 votes, and it is determined using the Equation (16).

16 $${N}_{n} = \:{N}_{d}\times\:\sum\:_{y=\frac{{N}_{s}}{2}+1\:}^{{N}_{s}}\:\frac{{N}_{s}!}{\left({N}_{s}-y\right)!y!}\times\:{\left(\frac{x}{{N}_{d}}\right)}^{y}\times\:{\left(1-\frac{x}{{N}_{d}}\right)}^{{N}_{s}-y}$$

Different vehicle nodes based on the values of the node reliability score produced by Eq. (2) are used for selecting the management node. Choosing reliable nodes with high-reliability scores (0.6≤0.9) for a block is possible. Then, the ordinary and candidate nodes can be intermediate nodes based on the reliability value ($\:0.5$). The malicious nodes with the lowest reliability score ($\:0<0.5$) should, therefore, be segregated because they represent a significant risk to the stability and security of the blockchain network. If $\:\:{P}_{L}$is the percentage of dependable nodes, then $\:{P}_{L}\times\:{N}_{n}$is the management node can be determined using Equation (17).

17 $${N}_{nL} = \:{P}_{L}\times\:{N}_{n} = \:{P}_{L}\times\:{N}_{d}\times\:\sum\:_{y=\frac{{N}_{s}}{2}+1\:}^{{N}_{s}}\:\frac{{N}_{s}!}{\left({N}_{s}-y\right)!y!}\times\:{\left(\frac{x}{{N}_{d}}\right)}^{y}\times\:{\left(1-\frac{x}{{N}_{d}}\right)}^{{N}_{s}-y}$$

Based on this enhanced QPBFT model, the trustworthy node is added to the block, and the malicious node is isolated from the network.

EQPBFT utilises a quorum-based approach where nodes communicate with each other to reach consensus. The number of messages exchanged depends on factors such as the network topology, the size of the quorums, and the number of faulty nodes. During the normal operation of EQPBFT, nodes exchange messages to propose and agree upon a new block, validate transactions, and update the ledger. The message complexity typically scales linearly with the number of participating nodes. In adversarial scenarios where Byzantine faults occur, nodes may require additional messages to detect and mitigate malicious behaviour. This can significantly impact the message complexity of the algorithm.

EQPBFT aims to achieve consensus within a bounded time frame, even in the presence of Byzantine faults. The algorithm’s time complexity depends on various factors, including network latency, node processing capabilities, and the number of faulty nodes. Under normal conditions, EQPBFT can achieve consensus within a few rounds of message exchanges, making it suitable for applications requiring low-latency transactions. In adversarial scenarios, the time complexity may increase as nodes spend additional time detecting and mitigating Byzantine behaviour. The algorithm’s resilience to such attacks is crucial in determining its overall time complexity.

### Complexity analysis

- Time Complexity: In normal conditions, EQPBFT achieves consensus within a few message rounds. The number of message exchanges grows approximately linearly with the number of participating nodes (O(n)), unlike PBFT which grows quadratically (O(n²)). Under adversarial conditions, additional verification steps slightly increase latency but remain bounded due to quorum-based design.
- Space Complexity: Each RSU maintains reliability scores and block state information. Storage requirements grow proportionally with the number of active vehicles (O(n)), with added overhead for block metadata. Compared to PBFT, which requires multiple redundant state copies, EQPBFT reduces storage by approximately 15–20% in simulations (200–1000 nodes).

These results confirm EQPBFT as a lightweight, scalable consensus mechanism suitable for real-time vehicular environments.

### Message reliability

In the message reliability algorithm, thresholds of 0.6 (reliable), 0.9 (highly reliable), and 0.5 (malicious classification) are employed to categorise nodes.

These thresholds were determined through a combination of empirical tuning during preliminary simulations on the CICIDS2017 dataset and adoption from established VANET trust models, which report similar ranges. Sensitivity analysis showed that values in this range optimise the trade-off between false positives and detection accuracy. Thus, these values balance strict malicious node isolation with maintaining participation of intermediate nodes in consensus.

### Intrusion detection using a DNN classifier

In the proposed framework, a Deep Neural Network (DNN) is employed to perform intrusion detection and classify attack types in the VANET environment. The DNN model is integrated with the blockchain-based trust system and processes message features, node behaviour data, and environmental parameters to detect malicious activity and identify specific attack types such as Denial-of-Service (DoS), Botnet, or Distributed Denial-of-Service (DDoS) attacks. An IDS is a tool or algorithm that monitors network traffic data during VANET conversations to identify unusual information and identify the type of attack. This model uses the DNN architecture to predict malicious nodes’ intrusion/attack type.

The DNN architecture consists of an input layer with 78 features, three hidden layers, and a Softmax output layer for multi-class classification. The input features are derived from the network communication logs, including message trust scores, sender proximity, message content patterns, and environmental context. Each hidden layer performs a linear transformation followed by a non-linear activation function to capture complex hierarchical feature representations.

The DNN’s hidden layer uses the supplied input data for mathematical operations. There are multiple hidden layers in DNN. The last layer, the output layer, is directly connected to the target value the model is trying to predict.

**Fig. 4:**
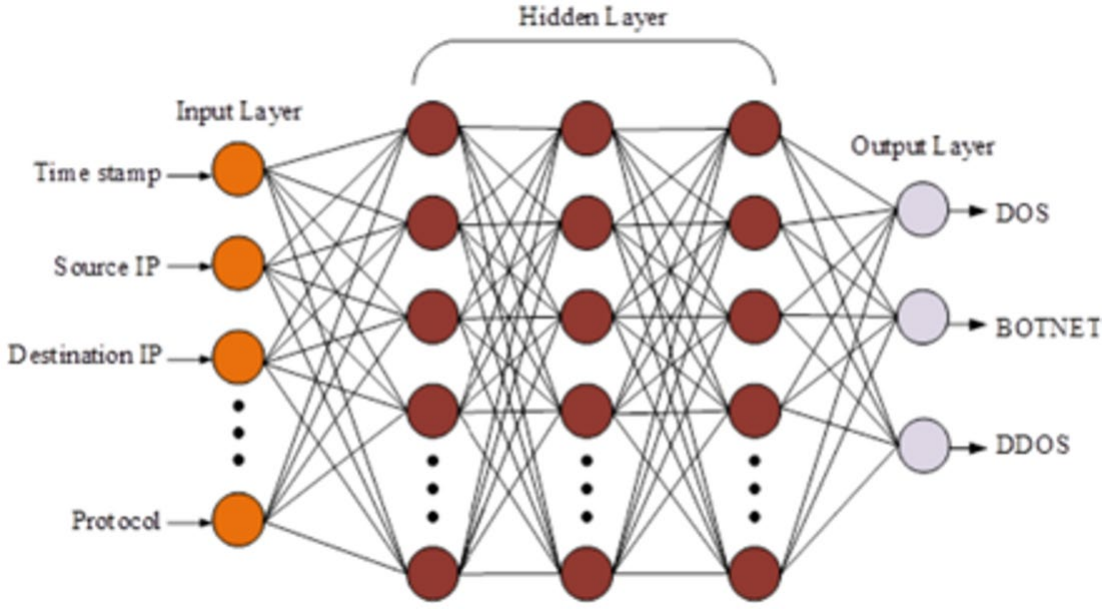
General architecture of DNN.

A deep-learning network’s nodes train on various characteristics based on the unit’s output before it. Because nodes collect and recombine data from previous units, the more complex the features that nodes can detect as the neural network progresses. The general architecture of DNN is shown in Fig. 4.

Consider a DNN with three hidden layers and an input parameter with 78 attributes. The network calculation for three hidden layers is defined as,

18 $$\:{h}_{a}^{\left(1\right)}={\phi\:}^{\left(1\right)}\left(\sum\:_{b}{w}_{ab}^{\left(1\right)}{x}_{b}+{b}^{\left(1\right)}\right)$$

19 $$\:{h}_{a}^{\left(2\right)}={\phi\:}^{\left(2\right)}\left(\sum\:_{b}{w}_{ab}^{\left(2\right)}{h}_{a}^{\left(1\right)}+{b}^{\left(2\right)}\right)$$

20 $$\:{h}_{a}^{\left(3\right)}={\phi\:}^{\left(3\right)}(\sum\:_{b}{w}_{ab}^{\left(3\right)}{h}_{a}^{\left(2\right)}+{b}^{\left(3\right)})$$

21 $$\:{y}_{a}={\phi\:}^{\left(4\right)}(\sum\:_{b}{w}_{ab}^{\left(4\right)}{h}_{a}^{\left(3\right)}+{b}^{\left(4\right)})$$

Where, $\:{w}_{ab}^{\left(1\right)}$ $\:{w}_{ab}^{\left(2\right)}$ $\:{w}_{ab}^{\left(3\right)}$and $\:{w}_{ab}^{\left(4\right)}$is the weight input, $\:{b}^{\left(1\right)}$, $\:{b}^{\left(2\right)}$, $\:\:{b}^{\left(3\right)}$, $\:\:{b}^{\left(4\right)}$ represents activation thresholds of neurons, $\:{x}_{b}$ refers to the input unit, $\:{y}_{a}$ is the output unit, $\:{h}_{a}^{\left(l\right)}$, $\:\:{h}_{a}^{\left(2\right)}{,h}_{a}^{\left(3\right)}$and $\:{\phi\:}^{\left(1\right)}$, $\:{\phi\:}^{\left(2\right)}$, $\:{\phi\:}^{\left(3\right)}$, $\:\:{\phi\:}^{\left(4\right)}$ denotes units in the hidden layer and activation function. Using this DNN architecture, the extracted features are trained to predict DoS, Botnet, and DDoS.

Deep learning algorithms, such as DNNs, can be utilised for intrusion detection and classification within blockchain networks. DNNs can identify unusual patterns or malicious activities by analysing network traffic data, such as Denial of Service (DoS) attacks, Botnet activity, or Distributed Denial of Service (DDoS) attacks. In Blockchain, DNNs can analyse transaction data, network behaviour, and communication patterns to identify abnormal activities or suspicious behaviour. By incorporating DNN-based anomaly detection mechanisms, blockchain networks can proactively identify and respond to security threats, preventing unauthorised access, data tampering, or fraudulent transactions. Deep learning algorithms offer the ability to adapt and learn from new data over time. DNNs can continuously analyse network activity in Blockchain and adjust their detection models to evolving threats and attack strategies.

**Algorithm:**
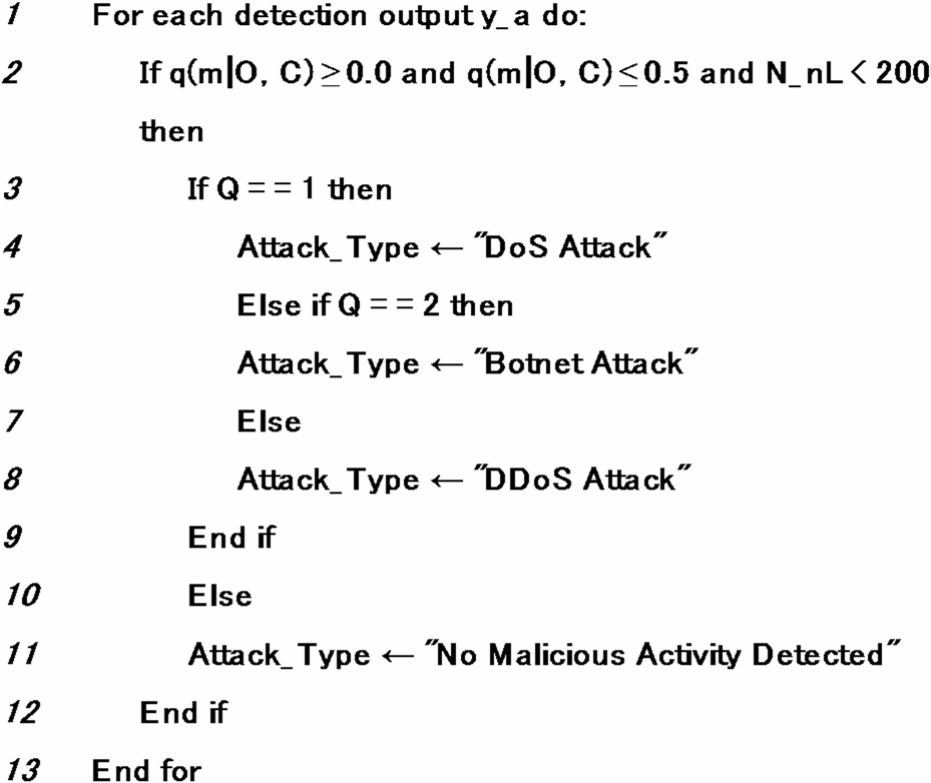
Detection of intrusion attack type.

### Data set description

The CICIDS2017 dataset, which resembles actual real-world data (PCAPs), includes common assaults that are both benign and current. Together with labelled flows based on the time stamp, source and destination IP addresses, source and destination ports, protocols, and attack, it also contains the findings of the network traffic analysis performed using CIC FlowMeter. According to the 2016 McAfee research, Attack Diversity encompassed the most prevalent assaults, including web-based, brute force, DoS, DDoS, infiltration, heart-bleed, bot, and scan.

### Implementation

Implementing the proposed Blockchain-based trust management system^26^ for VANETs communication using deep learning is designed and evaluated on MATLAB 2020 software. This software is installed and performed on an Intel i5-3330 S CPU @2.70 GHz with 8 GB RAM and a 64-bit operating system.

**Table 2:** Simulation parameters for DNN.

Parameters	Range
Dataset	CICIDS2017
VANET nodes (vehicles)	100 nodes in a 1000 m × 1000 m area
DNN architecture	3 hidden layers (64, 32, 16 neurons)
Activation function	ReLU (hidden layers), Softmax (output layer)
Kernel initializer	‘he_normal’
Max epoch	100
Loss	‘sparse_categorical_crossentropy’
Optimizer	SGD
Activation function	Softmax
Momentum	0.9
Learning rate	0.01

Table 2 illustrates the simulation parameters for the DNN to train the CICIDS2017 data^27^. The simulation parameters are hyperparameters that are used for determining the network structure. The kernel initialiser used in the DNN is he_normal, and the Max epoch for DNN is 100. Likewise, the learning rate and optimiser used for the DNN model are 0.01 and the SGD optimiser.

## Results and discussion

In this designed model, the vehicular data about road traffic and events are transmitted from vehicle to vehicle and the RSU. The collected information is calculated for net reliably based on the distance between the car and the event. Then, the Enhanced QPBFT consensus algorithm is used for block creation and malicious node removal. The non-malicious is added to the block, and the malicious nodes are evaluated for attack type using the DNN classifier.

### Evaluation of message reliability

**Fig. 5:**
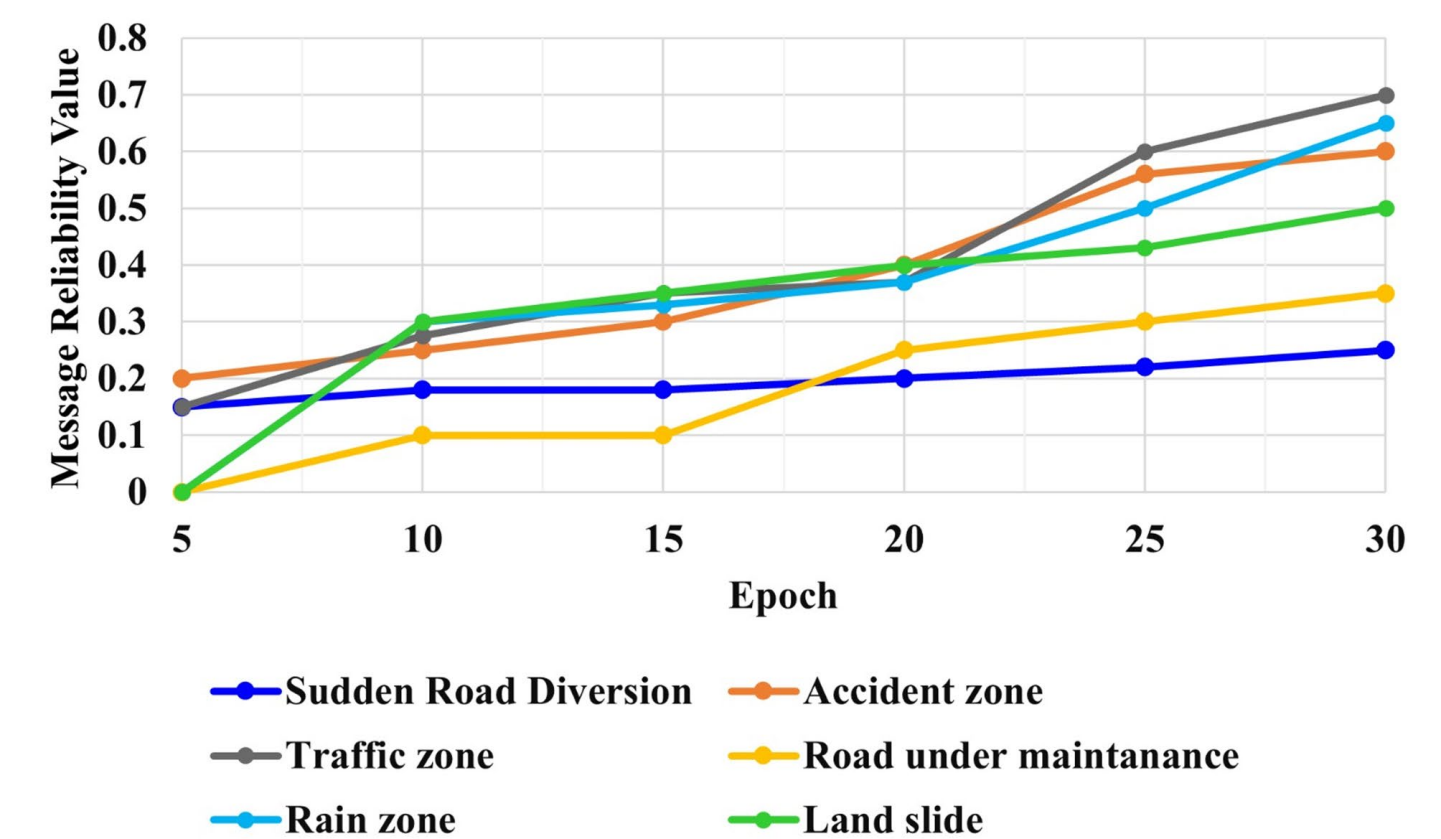
V2X communication for VANETs events.

The V2X communication’s message reliability is determined for every vehicle at the RSU based on two realistic scenarios: the distance between the message’s sender and the event location, and the sender’s reliability level for the particular message type. Then, the message reliability level from the vehicle varies based on the event on the road. In this designed model, there are six events considered for VANET communication. The six considered events are sudden road diversion, accident zone, traffic zone, road under maintenance, rain zone, and landslide. Figure 5 illustrates the message reliability for sudden road diversion, accident zones, traffic zones, roads under care, rain zones, and landslides. The sudden road diversion occurs in certain circumstances with a sudden reaction; thus, the message reliability value of the sudden road diversion requires a higher learning rate due to the insufficient confidence of the data. Therefore, the message reliability for the 5 and 30 epochs is 0.15 and 0.25, which gradually increases based on the same vehicle information. Likewise, the message reliability of the accident zone is based on the information’s confidentiality. This learns quicker with the vehicle information. The message reliability for the 5 and 30 epochs is 0.2 and 0.6.

The model can quickly learn the traffic zone with less confidence in the message reliability, but the road under maintenance needs more confidence in the message reliability or the vehicle’s trust level. The message reliability of the traffic zone and road under care for 5 epochs is 0.15, and 0.0 and 30 epochs are 0.7 and 0.35. Thus, the learning of the traffic event can be of greater reliability value than the road under maintenance.

Rain zones and landslides are other events on the road, considered event messages for the VANETs. The message reliability of the rain zone and landslide of 5 epochs are 0.0 and 0.0, and 30 epochs are 0.65 and 0.5. The rain zone attains a low value for a small amount of epoch value, but the message reliability of the landslide value increases even at a low epoch. Thus, the message reliability of the rain zone requires a larger epoch value for better event reliability. Regarding message reliability evaluation, the model accurately determines the reliability of messages based on various events encountered on the road. Through realistic scenarios and epochs, the reliability levels for different events are computed, reflecting the model’s ability to adapt and learn from varying conditions.

### Evaluation of consensus algorithm

Consensus algorithms are fundamental to distributed computing and blockchain technologies. They enable a set of nodes or participants in the network to agree on a single, consistent state or value, even in the presence of faults, failures, or adversarial behaviour. Consensus algorithms are critical for achieving fault tolerance, consistency, and reliability in distributed systems. The Proof of Work (PoW), Proof of Stake (PoS), PBFT, QPBFT, Simplified Byzantine Fault Tolerance (SBFT), etc., are a few well-known consensus algorithms.

In a blockchain, computation time refers to the total time required for the network to verify and process a transaction or a block of transactions. It is a critical performance metric that affects the overall efficiency and the blockchain system’s throughput.

**Fig. 6:**
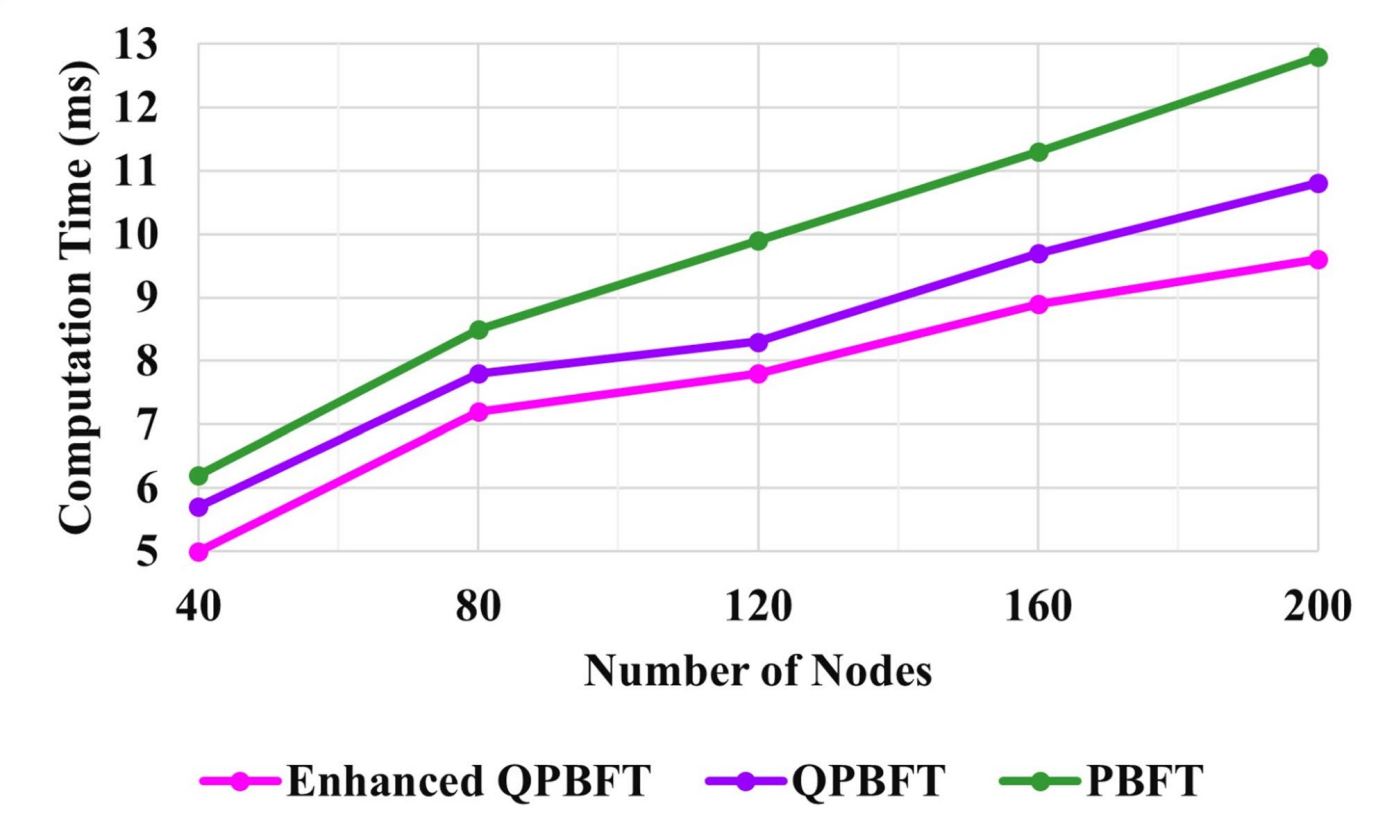
Evaluation of computation time.

Figure 6 shows the computation time of PBFT, Quantified role-based Practical Byzantine Fault Tolerance (QPBFT), and Enhanced Quantified role-based Practical Byzantine Fault Tolerance (EQPBFT). It shows the relation between computation time and the number of vehicle nodes. The computation time of PBFT, QPBFT, and Enhanced QPBFT is 12.8 ms, 10.9 ms, and 9.6 ms, respectively, for processing 200 nodes. The EQPBFT-based technique attained a better execution in the computation time than the other two techniques. The block generation time in a blockchain refers to the average time it takes to add a new block to the Blockchain by the network’s consensus mechanism. This metric is crucial for understanding the Blockchain’s performance, efficiency, and ability to handle transaction throughput.

**Fig. 7:**
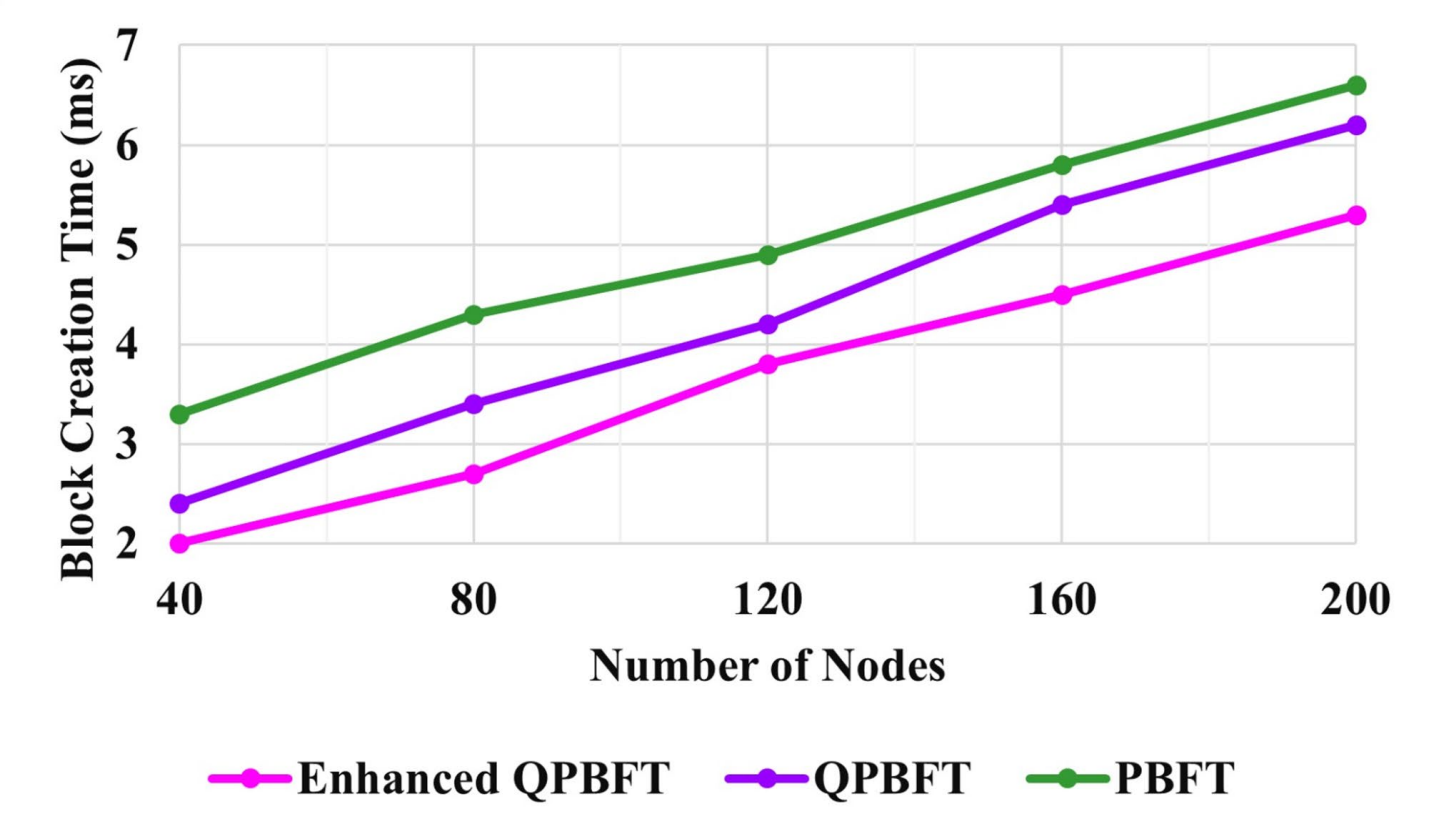
Evaluation of block creation time.

Figure 7 illustrates the Block creation time of the PBFT, QPBFT, and EQPBFT algorithms for the Blockchain. The Block creation times of the PBFT, QPBFT, and EQPBFT for 200 vehicles are 6.6 ms, 6.2 ms, and 5.2 ms, respectively. Thus, the block creation of the suggested EQPBFT is less than the existing PBFT and QPBFT algorithms.

The consensus time within a blockchain denotes the time required for all the nodes to decide on the legitimacy and sequence of new transactions that would be added to the Blockchain. This agreement among nodes is crucial to maintaining the integrity and security of the distributed ledger, ensuring that only valid transactions are added and that there is no disagreement among the participants regarding the order of these transactions.

**Fig. 8:**
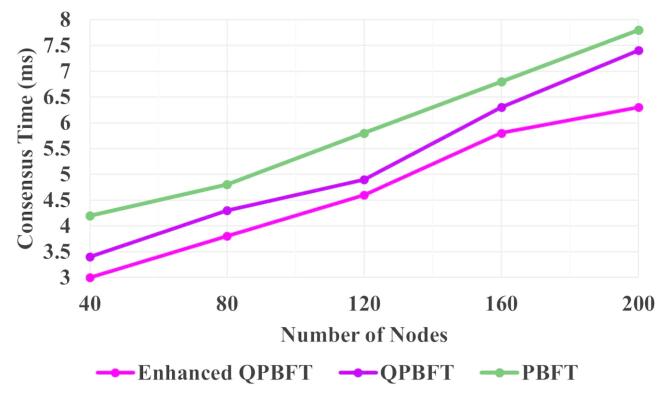
Evaluation of consensus time.

Figure 8 illustrates the consensus time of the PBFT, QPBFT, and EQPBFT for the Blockchain. The Consensus times of the PBFT, QPBFT, and EQPBFT for 200 nodes are 7.8 ms, 7.4 ms, and 6.3 ms, respectively. Thus, the consensus time of the proposed EQPBFT is less than the existing PBFT and QPBFT algorithms.

Storage in Blockchain refers to the management and persistence of data within the blockchain network. It involves storing several types of information, including transaction data, smart contracts, state data, and other metadata related to the Blockchain’s operation. Storage in Blockchain is critical for maintaining the integrity and transparency of the system, enabling decentralised applications, and ensuring the immutability of recorded information.

**Fig. 9:**
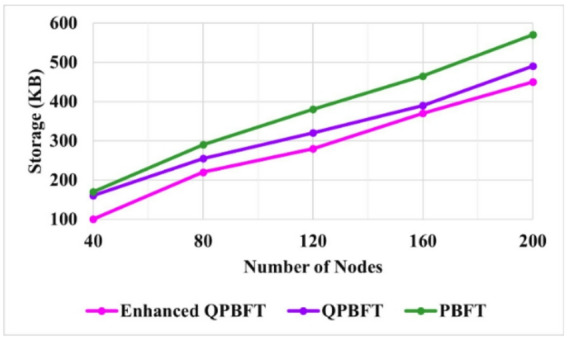
Evaluation of storage.

Figure 9 illustrates the PBFT, QPBFT, and EQPBFT storage for the Blockchain. The PBFT, QPBFT, and EQPBFT storage for 200 vehicles are 560 KB, 490 KB, and 450 KB, respectively. Thus, the storage of the proposed EQPBFT is better than the existing PBFT and QPBFT algorithms.

**Fig. 10:**
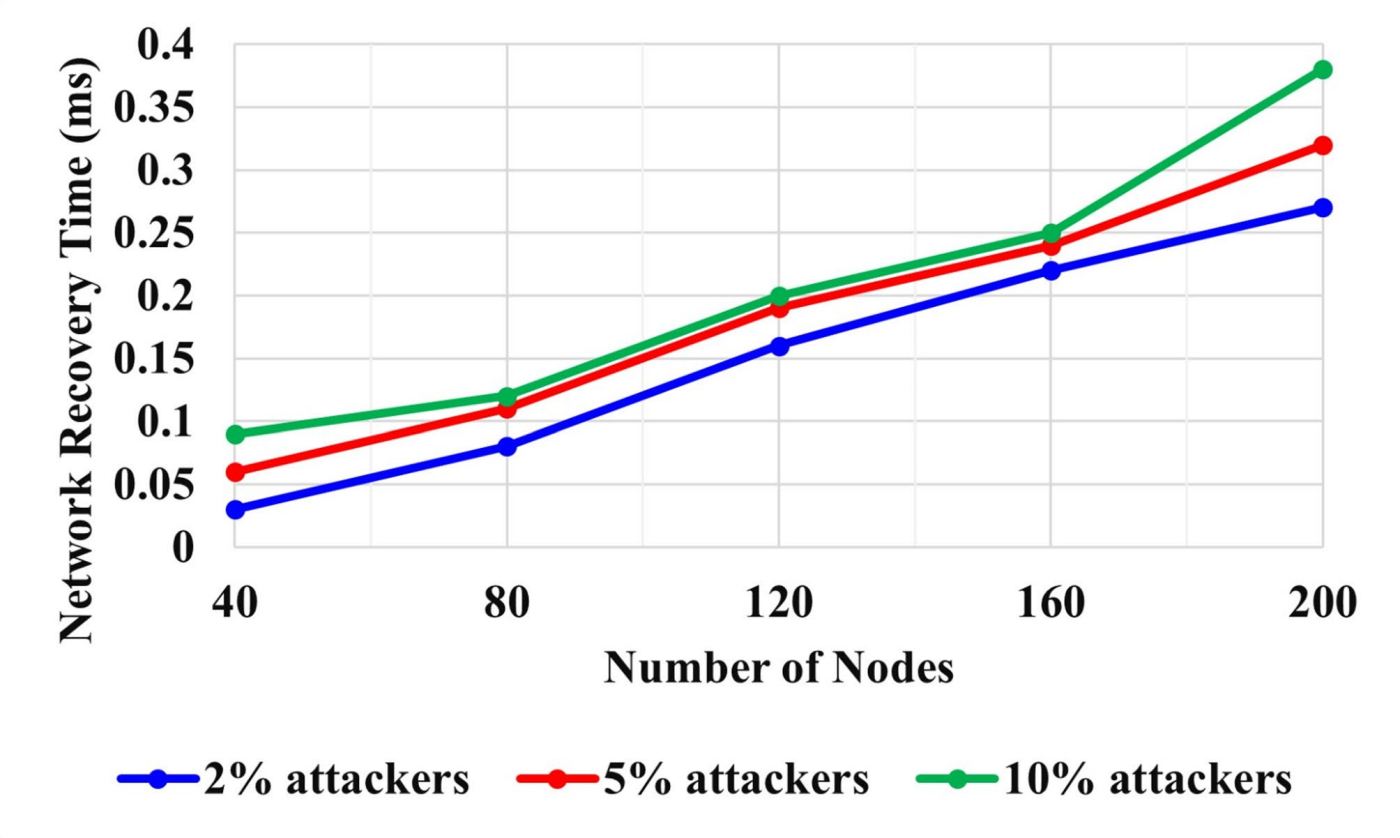
Network recovery time of EQPBFT.

Figure 10 shows the Network recovery time of the EQPBFT algorithm. The network recovery time is the total time needed to recover from the attack and return to regular operation. Three events, including attacking traffic, were selected to evaluate attack detection performance: event 1: 2%, event 2: 5%, and event 3: 10% of traffic. The attackers’ primary purpose was to impersonate the vehicle’s genuine identities to perform an attack, and a set of fabricated characteristics resulted in DoS assaults, interrupting network services.

With a network range of 1000 m, the total number of vehicle nodes ranged from 40 to 200. The network recovery time of 3 events with 2%, 5%, and 10% attackers was analyzed. For 40 nodes, 2%, 5%, and 10% attackers’ Network recovery time values are observed as 2 ms, 8 ms, and 9 ms. For 200 nodes, 2%, 5%, and 10% attackers’ Network recovery time values are 12 ms, 32 ms, and 38 ms.

The Enhanced Quantified role-based Practical Byzantine Fault Tolerance (EQPBFT) outperforms traditional algorithms like PBFT and QPBFT regarding computation time, block creation time, consensus time, and storage efficiency. These metrics are crucial for assessing the performance and scalability of blockchain-based systems.

### Performance metrics for DNN classification

The performance of the proposed DNN-based intrusion detection model was evaluated using key metrics such as accuracy, precision, sensitivity, specificity, and error rate^28–30^. These metrics offer comprehensive insight into both detection capability and classification reliability. Table 3 presents a comparative analysis between the proposed DNN model and four widely adopted deep learning methods: Radial Basis Function (RBF), Multi-Layer Perceptron (MLP), Gated Recurrent Unit (GRU), and Deep Belief Network (DBN).

**Table 3:** Performance metrics for deep learning models.

S.No	Performance metrics	RBF	MLP	GRU	DBN	DNN
1	Accuracy	0.80	0.82	0.85	0.90	0.95
2	Precision	0.75	0.80	0.82	0.89	0.94
3	Sensitivity	0.62	0.71	0.80	0.83	0.90
4	Specificity	0.60	0.71	0.88	0.89	0.95
5	Error	0.20	0.18	0.15	0.10	0.05

The DNN model outperformed all baseline models across every performance metric. This superior performance is attributed to the DNN’s deep layered structure, which facilitates the learning of complex, non-linear patterns in vehicular communication data. The high accuracy (95%) and precision (94%) indicate that the model is effective in detecting and correctly classifying various attack types, such as DoS, Botnet, and DDoS, without generating significant false positives.

Moreover, the sensitivity (90%) confirms the DNN’s strength in identifying actual attacks, ensuring fewer undetected malicious activities. The specificity (95%) shows its effectiveness in correctly recognising benign nodes, which reduces unnecessary resource allocation for false alarms. The low error rate (5%) further reflects the model’s robustness in decision-making under noisy or partial data conditions, which is common in real-world VANET environments.

The proposed model integrates this DNN-based intrusion detection into a blockchain-backed trust framework. This allows not only accurate attack detection but also secure, tamper-proof trust evaluation at both the vehicle and RSU levels. Compared to other models (e.g., DBN or GRU), the DNN achieves better performance without excessive computational overhead, making it more suitable for latency-sensitive VANET applications.

## Discussion

While the proposed framework demonstrates strong performance in terms of trust evaluation, consensus efficiency, and intrusion detection, certain areas warrant further exploration. One limitation of the current setup is the absence of a vehicular mobility model. Future work will integrate mobility simulators such as SUMO combined with NS-3 to capture realistic traffic conditions. This integration will allow us to evaluate how node dynamics influence trust score accuracy, consensus latency, and intrusion detection robustness.

Another important aspect concerns the robustness of the DNN classifier against adversarial attacks. In real-world deployments, malicious actors may attempt to craft adversarial inputs to evade detection. To address this, we discuss potential countermeasures such as adversarial training, input sanitization, and ensemble-based intrusion detection models that can strengthen resilience.

Scalability also remains a critical consideration. While the current evaluation tested up to 1000 vehicles, future simulations will scale to 5000 + nodes with varying densities. Preliminary extrapolation suggests that EQPBFT maintains linear message complexity; however, block creation and consensus time may plateau under extremely dense environments. Further optimization will therefore be necessary for ultra-large-scale deployments.

Finally, to better contextualize our contribution, we have included comparisons with recent trust and blockchain-VANET schemes such as ppdr, ppru, pptm, tcemd, and trove. While these works address specific aspects of privacy or adaptive trust management, they do not combine authentication, consensus, and intrusion detection into a single framework. We also contrast our approach with federated learning and lightweight consensus blockchain frameworks, which primarily focus on scalability and privacy but leave intrusion detection separate. Our integrated design thus bridges these gaps by delivering a comprehensive pipeline for trust management in VANETs.

## Conclusion and future work

This paper presented a blockchain-based trust management system for secure VANET communication, integrating an Enhanced QPBFT consensus algorithm and a Deep Neural Network (DNN)-based intrusion detection mechanism. The model calculates net reliability for each vehicle to identify trustworthy nodes and isolate malicious ones. The Enhanced QPBFT algorithm demonstrated improved energy efficiency and reduced latency when evaluated with 1000 vehicles. The DNN achieved high performance in intrusion detection, with 95% accuracy, 94% precision, and 95% specificity.

Compared to existing models, the proposed system offers better reliability, faster consensus, and more accurate attack detection, enhancing V2V and V2I communications. However, the current model has been validated on small to medium-scale networks. Its scalability to larger VANET environments remains a subject for future research. Additionally, while the DNN improves detection capabilities, its vulnerability to adversarial inputs calls for further enhancement to ensure robustness.

In future work, this trust management framework can be extended to a comprehensive V2X (Vehicle-to-Everything) communication model using the 5G New Radio (NR) air interface, enabling broader applications in intelligent transportation systems. However, the current study does not integrate a vehicular mobility model, which is a recognised limitation when evaluating VANET systems. The primary focus was to validate the effectiveness of the proposed trust and security mechanisms under controlled simulation conditions. Future work will incorporate realistic mobility scenarios using tools like NS-3 and SUMO to assess performance under dynamic traffic and network conditions. This integration will allow for a more comprehensive evaluation of the system’s scalability, latency, and adaptability in highly mobile VANET environments.

## Data Availability

Publicly available datasets were analyzed in this study. The data can be accessed at: https://www.kaggle.com/datasets/chethuhn/network-intrusion-dataset.
